# Integrated evaluation of the requirements and excretions of Cu, Fe, Zn, and Mn for broilers via a uniform design method

**DOI:** 10.3389/fvets.2023.1132189

**Published:** 2023-05-15

**Authors:** Kai Qiu, Zhimin Chen, Wenhuan Chang, Aijuan Zheng, Huiyi Cai, Guohua Liu

**Affiliations:** The Key Laboratory of Feed Biotechnology of Ministry of Agriculture, Institute of Feed Research, Chinese Academy of Agricultural Sciences, Beijing, China

**Keywords:** Cu, Fe, Zn, and Mn, trace element excretion, growth performance, interaction, broilers

## Abstract

This study aimed to determine the ideal balance profile of Cu, Fe, Zn, and Mn for broilers of 1–21 days of age via a uniform experimental design. In Experiment 1, 900 1-day-old Arbor Acres male broilers were randomly allotted to 15 dietary treatments with six replicates of 10 birds. A total of 14 experimental diets were formulated with the supplementation of 8~16, 123~160, 40~80, and 60~120 mg/kg of Cu, Fe, Zn, and Mn, respectively, in the basal diet, according to the uniform design method. The excretion of Cu, Fe, Zn, and Mn in the manure and the broiler performance were determined to build the ideal balance profile of these elements. Experiment 2 was conducted based on the ideal balance profile built in Experiment 1, to test its practicability using 720 broilers with two treatments. The dietary concentrations of Cu, Fe, Zn, and Mn in the control group were 15.19, 203.08, 76.78, and 86.13 mg/kg, respectively. In Experiment 1, the concentrations of Cu, Fe, Zn, and Mn in the diets were 16.96, 166.66, 46.01, and 60.26 mg/kg, respectively, when the average daily gain reached the optimum value. When the dietary concentrations of Cu, Fe, Zn, and Mn were 8.54, 130.66, 38.19, and 64.07 mg/kg, respectively, the total excretion of Cu, Fe, Zn, and Mn got the minimum value. There are corresponding ideal balance profiles for minimum excretion of a certain element. In Experiment 2, the dietary levels of Fe, Zn, and Mn were decreased by 17.93%, 40.08%, and 30.04%, respectively, which had no significant effect on average daily gain, average daily feed intake, and feed gain for 1~21 day-old broilers but markedly decreased the excretion of Cu and Mn and total excretion. It was concluded that there is a dilemma between growth performance and mineral excretion. Although dietary levels of Cu, Fe, Zn, and Mn supporting optimal growth are higher than those for minimizing mineral excretion, supplementing too many trace elements in the diets of broilers is unnecessary.

## 1. Introduction

Mineral trace elements, such as copper (Cu), iron (Fe), zinc (Zn), and manganese (Mn), play multiple crucial roles in the basic biochemical processes of animals (1, 2). Cu has been well-documented as a cofactor of many metalloenzymes involved in hydrolytic, electron transfer, and oxygen utilization reactions (3). Fe, as an ingredient of hemoglobin in erythrocytes, supports the transport system of oxygen in the blood (4). Zn works as the pivotal part of over 300 Zn-containing metalloenzymes, which participate in regulating physiological processes including antioxidant, anti-inflammatory, and immune responses, as well as apoptosis (5). Mn is an essential micronutrient that is required for the activity of a diverse set of enzymatic proteins and is involved in several significant physiological processes, including development, reproduction, immune function, energy metabolism, and antioxidant defenses (6). Given the importance of trace minerals, in the past century, nutritionists have paid more attention to the prevention of trace element deficiency. In addition, Cu and Zn with supra-nutritional levels were reported to promote growth performance, which consequently caused the abuse of mineral trace elements in the production of monogastric animals across the world (7–9). The excessive addition of trace elements to the diet results in large amounts of fecal discharge, which causes eutrophication and heavy metal pollution of soil and water (10, 11). Therefore, a better understanding of the balanced use of trace elements in feed is an essential component of sustainable livestock development and a direction for future research.

Considering nutrient waste and the environmental threat, the European Union has set lower dietary allowances for trace minerals in response to high levels in manure (12). In fact, many minerals have interactions in the body, which further makes the research more complicated (13, 14). Two types of relationships exist among trace minerals, antagonistic or synergistic, which occur at two stages, absorption and metabolism (15). Trace mineral interactions among Cu, Fe, Zn, and Mn are well demonstrated in broiler chickens (16, 17). A balanced nutrient supply between trace elements is essential to maintain an optimally functioning immune defense and performance (18). At present, the trace element requirements are mainly based on the studies of a single element (19, 20). However, antagonism between trace minerals at the absorptive stage results in inhibited absorption which means excess intake of a single element can decrease the intestinal absorption of other elements. Therefore, balancing the supply of trace elements has become the key point to minimize trace mineral excretion.

For a study with multiple parameters and interactions, uniform design is one of the methods to generate optimized experimental designs based on the design points being uniformly scattered on the experimental domain (21, 22). The objective of this research was to integrate the true requirements of Cu, Fe, Zn, and Mn for broilers to keep optimal performance while minimizing trace mineral excretion via the uniform design method.

## 2. Materials and methods

### 2.1. Animals and management

In Experiment 1, a total of 900 1-day-old healthy Arbor Acres male broilers were randomly allotted to 15 dietary treatments with six replicates in each. In Experiment 2, a total of 720 1-day-old healthy Arbor Acres male broilers were randomly allotted to two dietary treatments with 36 replicates in each. Both experiments lasted for 21 days.

Each replicate with 10 birds was raised in one stainless steel cage (size: 120 × 100 × 48 cm), with *ad libitum* access to diets and fresh water. The chicks were raised at 34 ± 1°C for 7 days and then gradually reduced to 25 ± 1°C by day 21, according to normal management practices. The birds were given 24 h lighting in the first 3 days and then 23 h lighting until day 21. The broilers were vaccinated for Newcastle disease and infectious bronchitis disease on days 1 and 7.

### 2.2. Experimental design and diets

A total of 15 combinations were chosen in random order according to DPS software (Data Processing System, v19.05) configuration for four factors (23). The combination of the four independent variables X_1_ (Cu), X_2_ (Fe), X_3_ (Zn), and X_4_ (Mn) at five variation levels is shown in Table 1. The concentrations of Cu, Fe, Zn, and Mn in the basal diet (8, 123, 40, and 60 mg/kg) were taken as the lowest dose, and the two times of National Research Council (1994) recommended contents were taken as the highest dose.

**Table 1:** Levels of Cu, Fe, Zn, and Mn in the dietary treatments.

**Factors**	**Dietary treatments**														
	1	2	3	4	5	6	7	8	9	10	11	12	13	14	15
Cu	8	16	14	10	14	14	16	16	12	10	10	12	12	8	8
Fe	123	153	133	153	143	160	123	133	143	123	143	160	133	160	153
Zn	40	80	70	70	60	80	50	40	40	80	60	70	50	60	50
Mn	60	120	60	75	90	105	120	75	105	90	60	120	90	75	105

In Experiment 1, the basal diet was a corn–soybean meal diet containing Cu, Fe, Zn, and Mn at 8, 123, 40, and 60 mg/kg diet (dry matter basis), respectively. The other nutrient levels in the basal diet were identical to the recommendations by China NY/T 33 (2004). Cu, Fe, Zn, and Mn were added in inorganic forms, and the contents were 25.22%, 30.11%, 34.39%, and 31.89%, respectively. The inherent contents of Cu, Fe, Zn, and Mn in the basal diet were 7.5, 123.0, 37.0, and 26.5 mg/kg, respectively. The ingredients and nutrition levels of the basal diet are presented in Table 2. As for Experiment 2, in order to verify the optimum integration and commercial feasibility using more birds, the dietary Cu, Fe, Zn, and Mn levels in the control group were 15.19, 203.08, 76.78, and 86.13 mg/kg, respectively. The diet for the experimental group was formulated based on the optimum integration values from Experiment 1. All experimental diets were cold-pelleted feed.

**Table 2:** Ingredients and chemical composition of basal diets for broilers during days 1–21.

**Item**	**Growing phase (Day 1–21)**
**Ingredients, %**	
Corn	55.62
Soybean oil	0.57
Soybean meal GB_2_	32.95
Distiller dried grains with solubles (DDGS)	3.00
Corn gluten meal	3.00
Salt	0.30
Dicalcium	1.78
Limestone	1.39
L-lysine hydrochloride	0.47
DL-methionine	0.22
Choline chloride	0.20
Premix^a^	0.50
Total	100
**Chemical composition**	
Metabolic energy^b^, kcal/kg	2,950
Crude protein^c^, %	21.00
Lysine^b^, %	1.30
Methionine^b^, %	0.55
Threonine^b^, %	0.84
Tryptophan^b^, %	0.29
Calcium^c^, %	1.00
Valid phosphorus^c^, %	0.50

a Provided the following per kg of diet: vitamin A, 12,000 IU; cholecalciferol, 2,000 IU; vitamin E (DL-α-tocopheryl acetate), 20 IU; vitamin K3, 2.15 mg; riboflavin, 8.00 mg; pyridoxine, 4.5 mg; vitamin B12, 0.02 mg; calcium pantothenate, 26 mg; nicotinic acid, 68 mg; folic acid,1 mg; biotin, 0.20 mg; I, 0.6 mg; Se, 0.3 mg; choline chloride, 1,000 mg.

b Calculated values.

c Analyzed values.

### 2.3. Sampling

A metabolism trial of 3 days duration was conducted on days 18 to 20. On day 17, the dropping tray under the cage was cleaned at 8:00 a.m. On day 18, feathers in the excretion were cleaned with a blower, and then the excreta per replicate on the dropping tray was homogeneously mixed. The proportion of the sample from each of the 3 days should be equal. Then, the samples were put into a hot air oven and dried at 65 °C for 3 days. After the samples cooled down and naturally regained moisture for 1 day, they were dried for another day to a constant weight. Subsequently, the samples were ground and put into self-zipped polyethylene sachets for the detection of Cu, Fe, Zn, and Mn.

Once the experimental broilers died or were culled, they would be recorded and weighed in time. On day 21, following 12 h of fasting, the body weight (BW) and feed intake (FI) of all broilers were recorded per replicate. Then, average daily feed intake (ADFI), ADG, and feed/gain (F/G) per replicate during the 1–21-day period were calculated and corrected by the mortality of broilers.

### 2.4. Chemical analysis

The feed or excreta samples were weighed at 0.5 g into each of the 15 Pyrex digestion flasks. In total, 7 ml of nitric acid was added, and 30 min later, 1 ml of 30% hydrogen peroxide was added. The 15 flasks, all equipped with a reflux condenser, were placed into the automated carousel (24). The digestion procedure was as follows: 130°C, 10 min, 1,600 W; 150°C, 5 min, 1,600 W; and 180°C, 20 min, 1,600 W. Then, the samples were separately transferred to a triangular flask with deionized water, heated on the electric hot plate with 180°C to 1–2 ml, filtered, and then diluted to 25 ml with 1% HNO_3_ for the determination of Cu, Fe, Zn, and Mn by atomic absorption spectrophotometer (ZA3000, Hitachi, Tokyo, Japanese). In this study, the concentrations of minerals in all feed and excreta were the measured values.

Calcium (procedure 4.8.03; AOAC, 2000), phosphorus (procedure 3.4.11; AOAC, 2000), and amino acids [procedure 982.30 E (a, b, c); AOAC, 2007] in the basal diet were measured in accordance with the standard procedure of the Association of Official Analytical Chemists (AOAC).

### 2.5. Statistical analysis

The data in Experiment 1 were analyzed by the non-linear multiple regression model of DPS software (v19.05), using the replicates as the experimental units. A mathematical model was developed by the following second-order polynomial equation:


$$\begin{array}{c} \text{Y}=\beta_{0}+\sum \beta_{\text{i}}{\text{x}}_{\text{i}}+\sum \beta_{\text{ii}}\overset{2}{\underset{\text{i}}{\text{x}}}+\overset{\text{n}}{\underset{\text{i}}{\sum }}\beta_{\text{ij}}{\text{x}}_{\text{i}}{\text{x}}_{\text{j}}\\ \text{i}\neq \text{j};\text{i},\text{j}=\text{1},\text{2},\text{3}\ldots \end{array}$$

where Y is the predicted response; β_0_ is a constant; β_i_, β_ii_, and β_ij_ are linear coefficient, square coefficient, and cross coefficient, respectively; and X_i_ and X_j_ are the independent variables. X_1_, X_2_, X_3_, and X_4_ are the contents of Cu, Fe, Zn, and Mn in the diet, respectively. The coefficient of determination (*R*^2^) could be used as a verdict if there is a close agreement between experimental and predicted values of the trace element excretion concentration. The significance of each coefficient was determined using the Student's *t*-test and *p*-value. The coefficient was deemed significant when the *p*-value was <0.05, and the lower the *p*-value was, the more significance it indicated (25).

The data in Experiment 2 were analyzed by a one-factor ANOVA procedure of SPSS software for Windows (v19.0, SPSS Inc., Chicago, IL, USA). The data were shown as mean ± standard error (SE), and a *p*-value of ≤0.05 indicated a significant difference.

## 3. Results

### 3.1. Performance of broilers in Experiment 1

The effects of Cu, Fe, Zn, and Mn concentrations on ADG, ADFI, and F/G of broilers are shown in Table 3. The performance have been corrected using the mortality, which was not provided because only a very small number of broilers died or were culled, and there was no difference between the treatment groups. The growth performance of broilers in the experiment was not as good as that of commercial production, which may be caused by the stress of experimental operation. Based on the response value, the statistical functional relationship was established among the dependent variables for live performance. The results of non-linear multiple regressions are presented in Table 4.

**Table 3:** Effects of different combinations of Cu, Fe, Zn, and Mn on performance in 1–21-day-old broilers.

**Items**	**ADG**	**ADFI**	**F/G**
	**(g/day per bird)**	**(g/day per bird)**	**(g/g)**
1	31.76 ± 0.68	50.93 ± 1.36	1.58 ± 0.01
2	37.68 ± 1.10	53.68 ± 1.54	1.54 ± 0.03
3	38.58 ± 0.72	56.15 ± 1.96	1.54 ± 0.02
4	36.89 ± 0.29	56.43 ± 0.94	1.57 ± 0.02
5	35.84 ± 0.91	53.55 ± 1.85	1.53 ± 0.01
6	34.31 ± 0.75	55.55 ± 1.46	1.57 ± 0.01
7	35.40 ± 0.49	54.53 ± 1.32	1.54 ± 0.03
8	35.93 ± 0.41	53.99 ± 0.63	1.56 ± 0.02
9	35.68 ± 0.64	54.56 ± 1.19	1.56 ± 0.01
10	33.31 ± 0.49	54.42 ± 1.83	1.57 ± 0.02
11	39.63 ± 0.36	57.15 ± 1.87	1.54 ± 0.02
12	37.43 ± 0.94	58.33 ± 1.02	1.59 ± 0.01
13	32.86 ± 0.31	52.43 ± 1.13	1.54 ± 0.01
14	37.12 ± 0.88	55.74 ± 1.41	1.57 ± 0.01
15	35.42 ± 1.02	55.77 ± 1.12	1.48 ± 0.01

n = 6; Mean ± Standard Error (SE).

ADG, average daily gain; ADFI, average daily feed intake; F/G: feed/gain.

**Table 4:** Significance of regression coefficient for different indexes.

**Items**		**Equation regression item**													
		**X**_1_	**X**_2_	**X**_3_	**X**_4_	**X**_1_ **X**_2_	**X**_1_ **X**_3_	**X**_1_ **X**_4_	**X**_2_ **X**_3_	**X**_2_ **X**_4_	**X**_3_ **X**_4_	** ${\text{X}}_{1}^{2}$ **	** ${\text{X}}_{2}^{2}$ **	** ${\text{X}}_{3}^{2}$ **	** ${\text{X}}_{4}^{2}$ **
I	*T*	–	6.96	–	–	5.53	–	5.3	–	7.71	–	–	–	–	7.57
	*p*-value	–	<0.01	–	–	<0.01	–	<0.01	–	<0.01	–	–	–	–	<0.01
II	*T*	–	–	3.74	–	4.09	2.25	2.98	2.35	4.76	–	–	3.24	–	4.75
	*p*-value	–	–	<0.01	–	<0.01	0.03	<0.01	0.03	<0.01	–	–	<0.01	–	<0.01
III	*T*	–	2.23	5.78	21.69	4.4	3.82	–	–	–	–	–	–	–	–
	*p*-value	–	0.03	<0.01	<0.01	<0.01	<0.01	–	–	–	–	–	–	–	–
IV	*T*	9.01	3.93	8.36	–	7.31	3.47	–	2.2	–	8.84	–	–	–	8.45
	*p*-value	<0.01	<0.01	<0.01	–	<0.01	<0.01	–	0.05	–	<0.01	–	–	–	<0.01
V	T	3.19	3.46	–	4.6	7.55	–	–	–	8.33	–	4.39	–	–	5.52
	*p*-value	<0.01	<0.01	–	<0.01	<0.01	–	–	–	<0.01	–	<0.01	–	–	<0.01
VI	*T*	–	–	8.3	–	–	–	–	–	–	3.07	–	–	5.06	2.55
	*p*-value	–	–	<0.01	–	–	–	–	–	–	<0.01	–	–	<0.01	0.02
VII	*T*	7.28	3.45	–	–	–	2.65	–	4.71	–	–	5.51	4.18	5.54	24.71
	*p*-value	<0.01	<0.01	–	–	–	0.01	–	<0.01	–	–	<0.01	<0.01	<0.01	<0.01

Items I, II, III, IV, V, VI, and VII, respectively, represent the equation of ADG, ADFI, total excretion, and Cu, Fe, Zn, and Mn excretion; – represent that there was no regression item.

*p* < 0.05 means significant difference.

The regression equation of Cu, Fe, Zn, and Mn on ADG is expressed as follows:


(1) $$\begin{array}{c} Y=-1.610+0.400X_{2}+0.006X_{4}X_{4}+0.015X_{1}X_{2}\\ \;-0.023X_{1}X_{4}-0.005X_{2}X_{4} \end{array}$$

The goodness of the regression was checked by the coefficient of determination (*R*^2^), whose value (*R*^2^ = 60%) indicated that it was reasonable to use the regression model (Eq. 1) for analyzing the trend of the response. Based on the analysis of the optimal solution, when Cu, Fe, Zn, and Mn were 16.96, 166.66, 46.01, and 60.26 mg/kg, separately, the optimum value of ADG was 54.15 g/day.

The effect of dietary Cu, Fe, Zn, and Mn on ADFI could be expressed as follows:


(2) $$\begin{array}{c} Y=16.171+1.054X_{3}+0.002X_{2}X_{2}+0.006X_{4}X_{4}+0.0238X_{1}X_{2}\\ \;-0.025X_{1}X_{3}-0.022X_{1}X_{4}-0.005X_{2}X_{3}-0.005X_{2}X_{4} \end{array}$$

The goodness of the regression was checked by the coefficient of determination (*R*^2^), whose value (*R*^2^ = 44%) indicated reasonable use of the regression model (Eq. 2) for analyzing the trend of the response.

According to Eqs 1 and 2 and the analysis of the optimal solution, the maximal ADG could be obtained when the concentrations of Cu, Fe, Zn, and Mn in the diet were 16.96, 166.66, 46.01, and 60.26 mg/kg, respectively. The results indicate that the optimum integration of ADG and ADFI is different, and the F/G shows no significant difference among treatments. So, it was much more reasonable to use ADG to represent the performance. The optimal performance was obtained when Cu, Fe, Zn, and Mn in the diet were 16.96, 166.66, 46.01, and 60.26 mg/kg, respectively.

### 3.2. Excretion of trace elements of broilers in Experiment 1

The excretion concentrations of trace elements for 21-day-old broilers are shown in Table 5. The results of non-linear multiple regressions are presented in Table 4.

**Table 5:** The concentration of trace elements in excreta for 1–21-day-old broilers.

**Treatment**	**Cu (mg/kg)**	**Fe (mg/kg)**	**Zn (mg/kg)**	**Mn (mg/kg)**	**Total excretion (mg/kg)**
1	27.91 ± 1.38	6.17 ± 0.26	83.54 ± 0.26	186.29 ± 0.99	76.69 ± 0.90
2	53.58 ± 0.30	5.72 ± 0.35	208.60 ± 2.41	347.39 ± 2.09	153.74 ± 0.44
3	36.13 ± 0.15	5.09 ± 0.13	171.93 ± 5.85	171.50 ± 2.32	98.88 ± 3.21
4	27.94 ± 1.22	6.06 ± 0.31	172.99 ± 4.73	210.04 ± 4.14	104.37 ± 2.54
5	43.62 ± 0.70	5.19 ± 0.47	155.98 ± 2.62	277.43 ± 3.51	120.82 ± 0.72
6	50.80 ± 1.27	7.22 ± 0.01	206.29 ± 0.56	336.05 ± 2.36	149.85 ± 0.94
7	48.87 ± 1.07	6.58 ± 0.07	127.57 ± 0.17	366.32 ± 4.97	137.13 ± 1.19
8	54.40 ± 0.83	2.61 ± 0.25	89.15 ± 2.16	263.01 ± 4.10	102.29 ± 1.77
9	32.54 ± 0.20	6.78 ± 0.45	90.04 ± 2.46	369.60 ± 5.34	125.25 ± 2.15
10	26.53 ± 0.50	8.15 ± 0.76	200.69 ± 3.80	277.61 ± 0.82	134.54 ± 4.75
11	29.10 ± 1.69	9.75 ± 0.82	152.75 ± 3.45	219.93 ± 1.64	101.89 ± 1.27
12	41.47 ± 0.53	5.67 ± 0.74	189.90 ± 5.39	392.72 ± 1.14	154.37 ± 3.53
13	42.28 ± 0.31	5.95 ± 0.55	126.42 ± 2.10	275.09 ± 0.66	112.44 ± 0.75
14	36.02 ± 0.13	6.73 ± 0.48	162.23 ± 1.46	248.57 ± 3.45	113.39 ± 1.24
15	32.04 ± 0.08	4.54 ± 0.12	116.45 ± 0.35	332.20 ± 3.11	121.14 ± 0.73

n = 6; Mean ± Standard Error (SE). Total excretion represents the mean value of Cu, Fe, Zn, and Mn.

The regression equation of Cu, Fe, Zn, and Mn on the total excretion of trace elements is expressed as follows:


(3) $$\begin{array}{c} Y=-11.94-0.238X_{2}+1.485X_{3}+0.821X_{4}\\ \;+0.037X_{1}X_{2}-0.081X_{1}X_{3} \end{array}$$

The fitness of Eq. 3 was checked by the coefficient of determination *R*^2^, which was calculated as 91%. Less than 9% of the total variance could not be explained by the model. Based on Eq. 3, Fe, Zn, and Mn have a stimulatory effect on total excretion. The interaction between Cu and Fe and Cu and Zn has a noticeable effect on the total mineral excretion. According to the analysis of the optimal solution, when the predicted trace element content of Cu, Fe, Zn, and Mn in the dietary was 8.54, 130.66, 38.19, and 64.07 mg/kg, respectively, the total excretion of Cu, Fe, Zn, and Mn obtained the minimum value of 75.07 mg/kg.

The effect of Cu, Fe, Zn, and Mn on Cu excretion is shown as follows:


(4) $$\begin{array}{c} \text{Y}=-\text{46}.0\text{66}+\text{13}.\text{721}{\text{X}}_{\text{1}}+0.\text{839}{\text{X}}_{\text{2}}-\text{2}.\text{517}{\text{X}}_{\text{3}}-0.00\text{5}{\text{X}}_{\text{4}}{\text{X}}_{\text{4}}\\ \;-0.0\text{96}{\text{X}}_{\text{1}}{\text{X}}_{\text{2}}+0.0\text{41}{\text{X}}_{\text{1}}{\text{X}}_{\text{3}}+\;0.00\text{5}{\text{X}}_{\text{2}}{\text{X}}_{\text{3}}+0.0\text{14}{\text{X}}_{\text{3}}{\text{X}}_{\text{4}} \end{array}$$

The coefficient of determination *R*^2^ of Eq. 4 was 91%, indicating that 91% of the variability in the response could be explained by the model (Eq. 4). Equation 4 shows that Cu, Fe, and Zn in the diet have a positive effect on Cu excretion, and the interaction effects of Cu and Fe, Cu and Zn, Fe, and Zn, and Zn and Mn were significant. The analysis of optimal solution also shows that when the optimum conditions for Cu excretion amount were Cu, 8.3; Fe, 126.93; Zn, 90.9; and Mn, 58.13 mg/kg, respectively, in the diet, the predicted Cu excretion obtained the minimum value.

The effect of dietary Cu, Fe, Zn, and Mn on Fe excretion could be expressed as follows:


(5) $$\begin{array}{c} \text{Y}=-\text{8}.0\text{84}-\text{2}.\text{586}{\text{X}}_{\text{1}}+0.\text{151}{\text{X}}_{\text{2}}+0.\text{425}{\text{X}}_{\text{4}}-0.0\text{86}{\text{X}}_{\text{1}}{\text{X}}_{\text{1}}\\ \;+0.00\text{2}{\text{X}}_{\text{4}}{\text{X}}_{\text{4}}+0.0\text{31}{\text{X}}_{\text{1}}{\text{X}}_{\text{2}}-0.00\text{6}{\text{X}}_{\text{2}}{\text{X}}_{\text{4}} \end{array}$$

The coefficient of determination (*R*^2^) of Eq. 5 was 0.59%, indicating a close agreement between experimental and predicted values of the Fe excretion. The regression equation (Eq. 5) indicated that Cu, Fe, and Mn in the feed had a remarkable effect on Fe excretion, and the interaction effects of Cu and Fe and Fe and Mn were noteworthy. When the predicted Fe excretion obtained the minimum value, the trace element contents of Cu, Fe, Zn, and Mn in the diet were 18.14, 121.81, 55.61, and 58.86 mg/kg, respectively.

The relationship between Zn excretion and dietary trace element concentration is expressed by a second-order polynomial as follows:


(6) $$\begin{array}{c} \text{Y}=-\text{1}0\text{1}.\text{9}0\text{6}+\text{5}.\text{647}{\text{X}}_{\text{3}}-0.0\text{34}{\text{X}}_{\text{3}}{\text{X}}_{\text{3}}-0.00\text{4}{\text{X}}_{\text{4}}{\text{X}}_{\text{4}}\\ \;+0.0\text{13}{\text{X}}_{\text{3}}{\text{X}}_{\text{4}} \end{array}$$

The coefficient of determination *R*^2^ of Eq. 6, which was calculated to be 95%, indicates that only <5% of the total variance could not be explained by the model. Based on Eq. 6, only Zn had a conspicuous effect on the concentration of Zn excretion, and the interaction effects of Zn and Mn were positive. The analysis of the optimal solution indicates that when the Zn excretion obtained the minimum value (88.2 mg/kg), the trace element contents of Cu, Fe, Zn, and Mn in the diet were 12.68, 122.15, 41.42, and 62.23 mg/kg, respectively.

The regression equation of Cu, Fe, Zn, and Mn on Mn excretion is expressed as follows:


(7) $$\begin{array}{c} \text{Y}=\text{818}.\text{849}+\text{47}.\text{266}{\text{X}}_{\text{1}}-\text{14}.\text{4}0\text{7}{\text{X}}_{\text{2}}-\text{1}.\text{496}{\text{X}}_{\text{1}}{\text{X}}_{\text{1}}+0.0\text{66}{\text{X}}_{\text{2}}{\text{X}}_{\text{2}}\\ \;+0.0\text{57}{\text{X}}_{\text{3}}{\text{X}}_{\text{3}}+0.0\text{15}{\text{X}}_{\text{4}}{\text{X}}_{\text{4}}-0.\text{153}{\text{X}}_{\text{1}}{\text{X}}_{\text{3}}-0.0\text{47}{\text{X}}_{\text{2}}{\text{X}}_{\text{3}} \end{array}$$

The fitness of the model (Eq. 7) was also analyzed by the coefficient of determination *R*^2^, which was calculated as 95%, suggesting that the model was efficient and could explain 95% of the experimental data. The regression equation (Eq. 7) demonstrated that Cu and Fe in the diet have a positive effect on Mn excretion, and the interaction effects of Cu and Zn, as well as Fe and Zn, were prominent for Mn excretion. When the Mn excretion obtained the minimum value (155.85 mg/kg), the trace element contents of Cu, Fe, Zn, and Mn in the feed were 8.05, 140.68, 65.51, and 60.7 mg/kg, respectively.

The *p*-values of all the quadratic terms in Eqs 1–7 were below 0.05 (Table 4), indicating that the quadratic terms also had significant effects on the responses, and the effects of the variables on the responses were not a simple linear relationship. The interaction between elements was significant.

### 3.3. Performance and trace elements excretion of broilers in Experiment 2

In Experiment 2, compared with the control group, dietary levels of Fe, Zn, and Mn in the experimental group were decreased by 17.93%, 40.08%, and 30.04%, respectively, whereas it had no significant effect on the ADG, ADFI, or F/G of the broilers from 1 to 21 days (*p* > 0.05, Table 6). The excretion of Cu, Mn, and total trace elements was markedly decreased (*p* < 0.05, Table 7).

**Table 6:** Effects of different groups with different concentrations of trace elements on live performance for 1–21-day-old broilers.

**Items**	**ADG**	**ADFI**	**F/G**
	**(g/d per bird)**	**(g/d per bird)**	**(g/g)**
Control	33.8 ± 1.3	49.9 ± 2.2	1.48 ± 0.05
Treatment	33.9 ± 2.7	50.8 ± 1.1	1.51 ± 0.12
*p*-value	0.234	0.182	0.701

Mean ± Standard Error (SE).

**Table 7:** Effects of different groups with different concentrations of trace elements on excreta for 1–21-day-old broilers.

**Groups**	**Items**				
	**Copper (mg/kg)**	**Iron (mg/kg)**	**Zinc (mg/kg)**	**Manganese (mg/kg)**	**Total excretion (mg/kg)**
1	74.27 ± 1.98	314.78 ± 8.3	254.36 ± 4.27	317.38 ± 4.26	240.20 ± 3.78
2	63.78 ± 0.62	325.16 ± 20.54	245.21 ± 1.43	225.36 ± 7.17	214.88 ± 2.17
*p*-value	<0.01	>0.05	>0.05	<0.01	<0.05

Mean ± Standard Error (SE). Total excretion represents the mean value of Cu, Fe, Zn, and Mn.

## 4. Discussion

Many trace minerals such as Cu, Fe, Zn, and Mn are antagonistic or synergistic during their absorption in the gastrointestinal tract and metabolism in the body (15, 26). In addition to benefiting body health, a balanced intake of trace elements is expected to improve their bioavailability and decrease excretion (18, 27). Therefore, in order to mitigate the threat of trace element excretion to the environment, the optimal combination of Cu, Fe, Zn, and Mn in the diet of broilers is worth investigating.

The supplementation of dietary Chinese yam polysaccharide-Cu (0.10 g/kg) improved the growth, immunity, and oxidation resistance of broilers (28). Excess Cu induces hepatic cholesterol metabolism dysfunction, and the miR-455-3p-OXSR1 axis works as a regulator of autophagy under Cu stress (29, 30). Dietary Cu content ranging from 7 to 16 mg/kg could satisfy the requirement of broilers with ages from 1 to 21 days (31). The BW and feed conversion ratio did not differ when broiler chicks were fed the basal diet containing 0, 200, 400, or 600 mg/kg of Cu (32). In the present study, the optimum Cu concentration is 16.96 mg/kg. It indicates that a low concentration of Cu could satisfy the requirement of broilers and too high a concentration may not be needed. Dietary supplementation of ferrous glycinate, a chelating agent composed of glycine and Fe, improves intestinal barrier function by modulating microbiota composition in ducks (33). High-dietary organic Fe supplementation decreases growth performance and induces oxidative stress in broilers (34). A high dosage of Fe supplement in the feed diet can induce oxidative stress in Chinese Yellow broilers, and the composition of microbiota in the cecum changed (35). Further additions of Fe increasingly depressed the growth of broilers (36), which was confirmed again in the present study as weight gain responses rose to a plateau when 20–60 mg Fe was added in diets with the inherent 110 mg/kg Fe. In the current study, the optimum ADG was observed with 166.66 mg/kg Fe in diets. Overall, it shows that for the growth performance of broilers, the amount of Cu and Fe added to diets is not as high as it could be.

The meta-analysis reported that dietary Zn supplementation had a positive effect on the growth performance of broilers (9). Organic Zn could enhance the expression of related transporters in the jejunum and ileum of broilers (37). Inorganic Zn was a suitable immunomodulator of intestinal immunity for *Ascaridia galli*-infected chickens (38). Food-grade ZnO nanoparticle exposure effects were associated with supporting intestinal development (39). The previous report shows that supplementation of inorganic Zn and Mn in the diet at 40 and 60 mg/kg, respectively was sufficient for broilers (13). Dietary Zn at concentrations of 0, 20, 40, or 80 mg/kg did not alter the growth rate and feed conversion ratio of 1–20-day-old broilers (40). ADG, ADFI, and F/G of chicks were not influenced by Mn supplementation at 0, 20, 40, 60, 80, or 100 mg/kg to the basal diet (41). In addition, adding organic Mn also did not significantly improve ADG, ADFI, and F/G during any periods of growth (16). Nevertheless, some researchers suggest that a too low level of Zn and Mn in diets may cause leg problems, while high levels could promote growth but cause environmental pollution (42). Mn deficiency induced tibial dyschondroplasia of broiler chicks through the downregulation of HIF-1α (43). The application of organic Mn sources may be an effective way to reduce economic losses and resolve animal welfare concerns due to tibial dyschondroplasia in commercial poultry farming (44). Mn alleviates heat stress of primary cultured chick embryonic myocardial cells via enhancing manganese superoxide dismutase expression and attenuating heat shock response (45). Mn methionine hydroxyl analog-chelated dietary supplementation improved the growth performance and trace element deposition in broilers, and the optimum Mn-MHAC level to meet the Mn requirement of broilers is 50–75 mg of Mn/kg diet (46). In the current study, according to the optimum ADG, the values of Zn and Mn in the diet were 46.01 and 60.26 mg/kg, respectively, which are similar to the contents of them in the basal diet. It can be seen that there is little need to add additional Zn and Mn to the basic diet to promote the growth of broiler chickens.

The supplementation of Cu from 10 to 150 mg/kg and Zn from 50 to 300 mg/kg did not improve the growth performance, but Cu and Zn concentrations in excreta increased with the increase of dietary Cu or Zn levels (47). Cu excretion decreased by 35% when supplemental Cu was reduced from 12 to 4 mg/kg. Decreasing dietary Zn concentration from 120 to 40 mg/kg reduced zinc excretion by 50%. This can potentially decrease the accumulation of Cu and Zn in the environment without compromising the growth performance of broilers (32). During each excreta collection period, increased supplemental Zn concentration significantly increased Zn excretion (40). Adding organic Cu or Fe alone did not significantly improve bird performance and was mostly excreted (16). In the present study, it was confirmed again that the excretion of Cu, Fe, and Zn increases, respectively, with the increase in Cu, Fe, and Zn supplements in the diet. It can be deduced that Cu, Fe, and Zn cannot be deposited in the body, and excess amounts would be excreted. However, the concentration of Mn in the diet had no significant effects on the excretion of Mn in the current study, which may be because most of Mn can be deposited in the organization of broilers.

Interaction, especially antagonism among mineral elements, has been well-studied by nutritionists (13–17). Dietary Fe levels decreased Mn utilization in MnSO_4_-treated broilers but did not influence Mn utilization in MnLys-treated broilers. This was evaluated by Mn concentrations in the serum and heart, and the activity and mRNA expression of manganese-containing superoxide dismutase in the heart (48). In this study, there were some complex antagonistic actions for target element excretion among the four elements. Both Fe and Zn had significant effects on Cu excretion. The antagonism between Zn and Cu occurred when the inorganic forms of these two minerals were included in a chick diet. The underlying mechanism is unclear and may be due to the sequestration of dietary Cu by intestinal metallothionein induced by high dietary Zn content (49). Mn supplementation at 120 mg/kg or above in diets could significantly decrease Cu accumulation in broilers (13). In the present study, Mn supplementation has no effect on Cu excretion. The discrepancy may be due to the different amounts of Mn added to the diet because competition between elements with similar chemical characteristics and uptake by non-regulated processes only takes place at high intake levels. Fe and Mn share binding sites in the gut mucosa, and therefore are thought to compete with each other for absorption (50). It is consistent with our study that Fe supplementation increased the excretion of Mn in which excess Fe impairs Mn utilization. Cu and Fe are essential mineral elements that exhibit important interactions and possible competitive inhibitions in transport and bioavailability (51). In the present study, Cu supplementation also has a negative effect on Fe excretion. Systemic Cu deficiency would result in cellular Fe deficiency because of the capacity of Fe and Cu to participate in one-electron exchange reactions (52). This indicates that the use of Cu and Fe in the broilers would be mutually reinforcing.

## 5. Conclusion

There is a dilemma between growth performance and mineral excretion in broilers. The levels of mineral trace elements that support optimal growth performance are higher than those that minimize mineral excretion. Dietary supplements with 16.96, 166.66, 46.01, and 60.26 mg/kg of Fe, Cu, Zn, and Mn, respectively, in turn, are recommended for broilers. Antagonistic actions for target element excretion exist among the four elements. The total excretion of Cu, Fe, Zn, and Mn for broilers obtains the minimum value when the dietary concentration of Cu, Fe, Zn, and Mn was 8.54, 130.66, 38.19, and 64.07 mg/kg, respectively.

## Data availability statement

The raw data supporting the conclusions of this article will be made available by the authors, without undue reservation.

## Ethics statement

The animal study was reviewed and approved by Animal Ethics Committee of the Chinese Academy of Agricultural Sciences.

## Author contributions

GL and HC: conceptualization, writing—reviewing and editing, and funding acquisition. KQ, AZ, WC, and GL: methodology. KQ, ZC, and GL: data analysis. KQ: writing—original draft preparation. All authors have read and agreed to the published version of the manuscript.
